# Unusual case of exacerbation of sub-acute descending necrotizing mediastinitis

**DOI:** 10.1186/1471-2482-13-S2-S31

**Published:** 2013-10-08

**Authors:** Vincenzo Di Crescenzo, Paolo Laperuta, Filomena Napolitano, Chiara Carlomagno, Michele Danzi, Bruno Amato, Alfredo Garzi, Mario Vitale

**Affiliations:** 1 Department of Medicine and Surgery, University of Salerno, Italy; 2 Department of Clinical Medicine and Surgery, University of Naples "Federico II Italy", Italy

**Keywords:** mediastinitis, descending, necrotizing

## Abstract

Descending necrotizing mediastinitis is a life-threatening complication of an oropharyngeal infection that requires prompt and aggressive medical and surgical therapy. Herein, we report unusual case of man suffering of sub-acute mediastinal infection due to odontoiatric abscess which exacerbated at 3 months after its first presentation. Chest X-ray and CT scan demonstrated soft-tissue swelling of the neck and encapsulated fluid collections with gas bubbles within anterior mediastinum, especially on the right side. Bilateral anterior neck dissections were performed and blunt dissection, irrigation and debridement were carried out to several centimetres below the sternal manubrium. Then, right standard thoracotomy was performed with debridement of the anterior mediastinum. Four tubes were placed in the mediastinum and pleural cavity on the right side, and two tubes were placed in the left thoracic cavity. Follow-up CT scans of neck and chest showed the resolution of infection.

## Introduction

Descending necrotizing mediastinitis (DNM) is a life-threatening complication of an oropharyngeal infection that requires prompt and aggressive medical and surgical therapy. Delay of diagnosis and inappropriate drainage of the mediastinum are the main causes of mortality in this life threatening condition [1,2]. Herein, we report a case of man suffering of sub-acute mediastinal infection due to odontoiatric abscess which exacerbated at 3 months after its first presentation. Aggressive surgical treatment with left in mediastinum multiple drainage allowed the resolution of the infection.

## Clinical case

**Figure 1:**
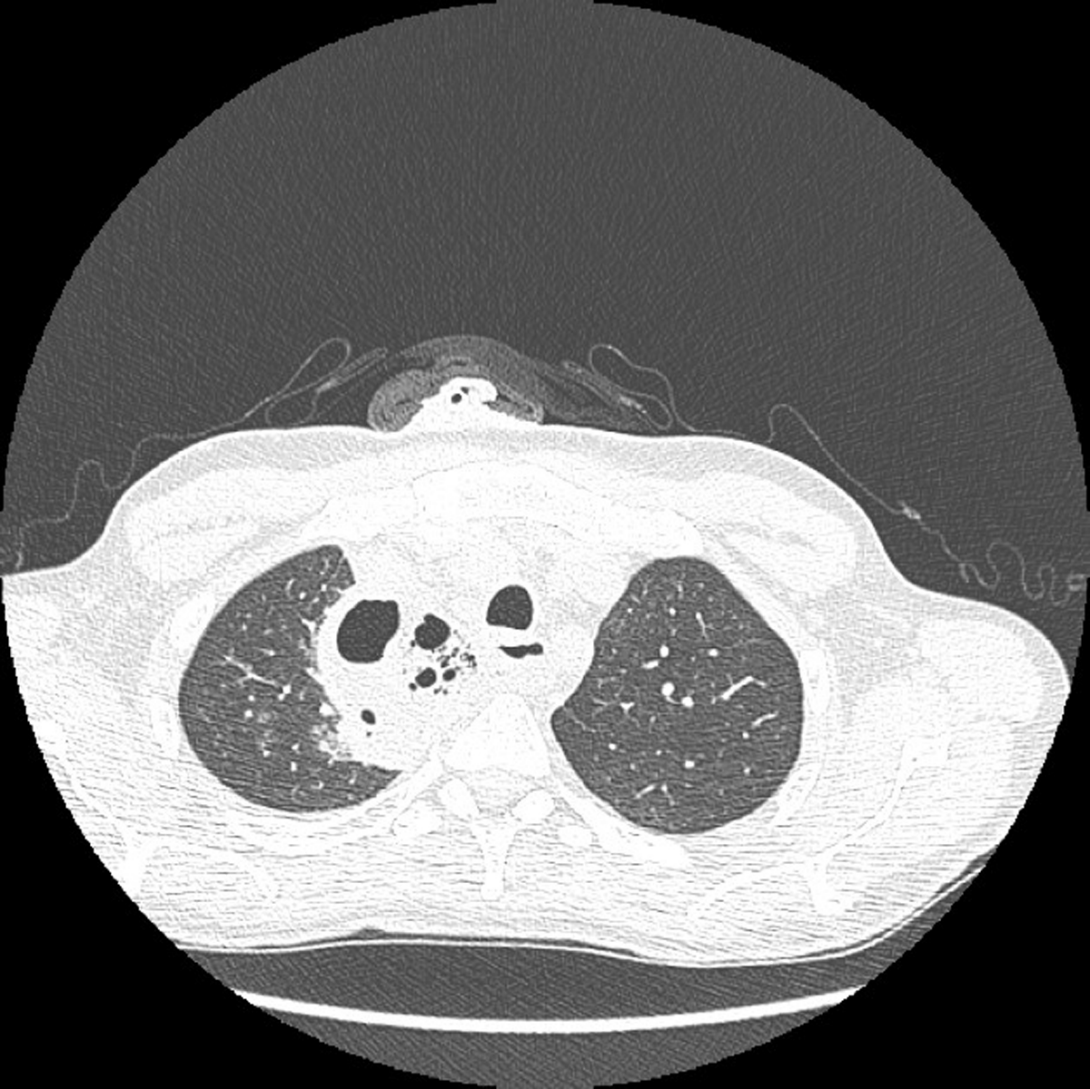
**CT scan demonstrated soft-tissue swelling of the neck and encapsulated fluid collections with gas bubbles within anterior mediastinum, especially on the right side**.

A 50 year-old man was admitted in our institution for neck pain, dysphagia, high-fever, and chest pain. Such symptoms began following infected foot ulcer 7 days before. She had a history of diabetes, and chronic obstructive bronchitis; yet, three months before she was admitted to a local hospital for mediastinal abscess as complication of odontogen abscess. However, the patient refused further investigations, and started antibiotic therapy for a time not better defined. On examination she had bilateral diffuse neck erythema, oedema, and induration. Broad-spectrum antibiotics were initiated empirically. Chest X-ray and CT scan demonstrated soft-tissue swelling of the neck and encapsulated fluid collections with gas bubbles within anterior mediastinum, especially on the right side (Figure 1).

**Figure 2:**
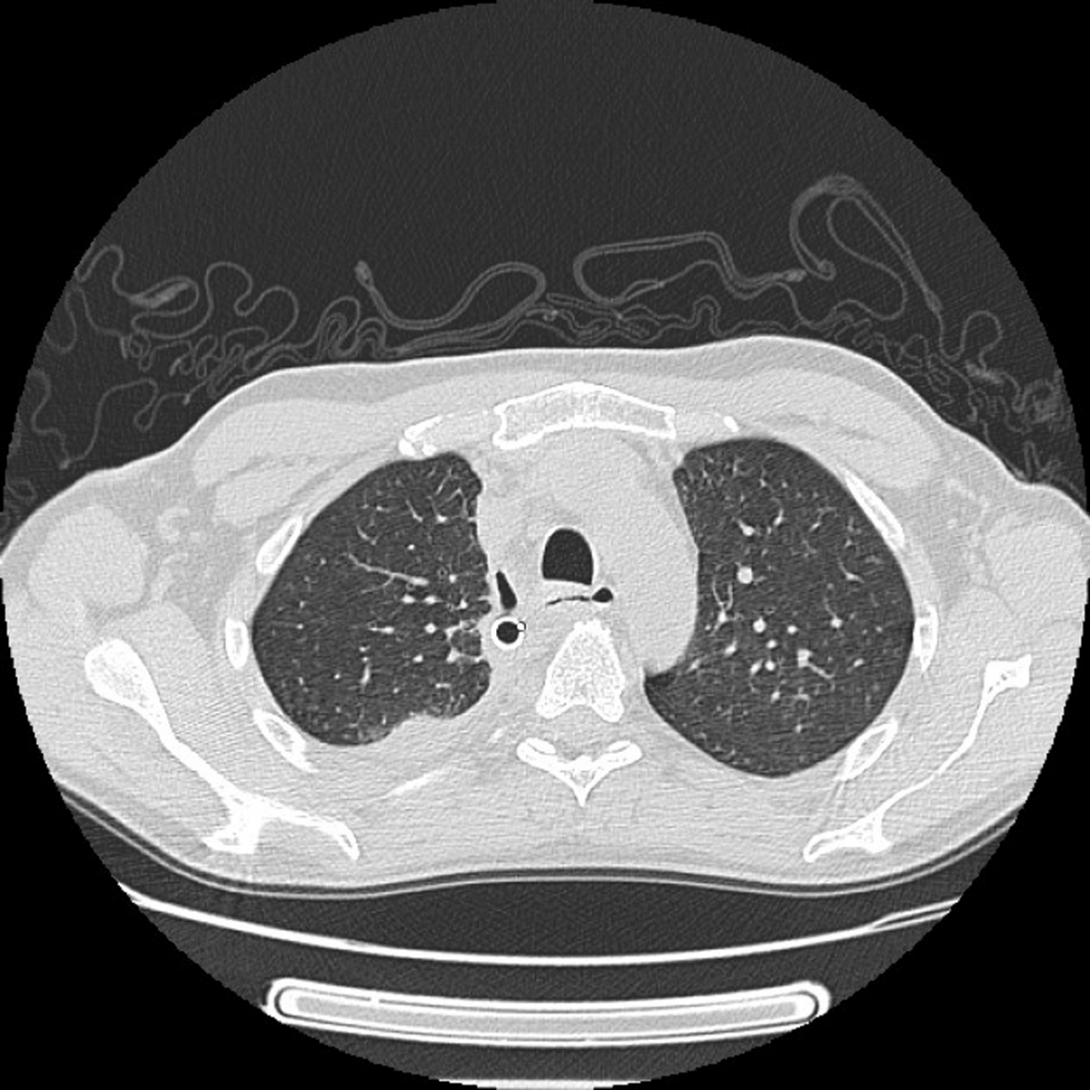
**CT scan demonstrated the resolution of infections**. It is well evident the chest drain within mediastinum on the right side that allowed the drainage of infection.

After review of the images, the patient was taken to the operating room. Bilateral anterior neck dissections were performed and blunt dissection, irrigation and debridement were carried out to several centimetres below the sternal manubrium. Then, right standard thoracotomy was performed with debridement of the anterior mediastinum. We irrigated the mediastinum and thoracic cavity with copious warm saline (approximately 5000-10,000 ml) during the operation. Four tubes were placed in the mediastinum and pleural cavity on the right side, and two tubes were placed in the left thoracic cavity. Bacteriologic results from materials obtained from the neck, pleura, mediastinum, pericardium, and blood revealed in all cases a polymicrobial infection, with mixed aerobic and anaerobic organisms. A repeated bronchoscopies were attended [3,4]. Follow-up CT scans of neck and chest showed the resolution of infection (Figure 2). He was discharged on post operatory day 21. Actually is well without symptoms.

## Discussion

The most dreaded and probably lethal form of mediastinitis is the diffuse necrotizing variety that occurs as a complication of infection of the oropharynx. The best term of such mediastinitis is DNM; descending because the infection uses fascial planes in the neck to gain access to the mediastinum, and necrotising because the infection is often polymicrobial and gas-producing [5]. Infections originating in the fascial planes of the head and neck spread downward into the mediastinum along the cervical fascias, facilitated by gravity, breathing, negative intrathoracic pressure, and the absence of barriers in the contiguous fascial planes of neck and mediastinum. DNM diagnosis implies the established relationship between mediastinitis, oropharyngeal infection, and radiological findings of mediastinitis. Delay of diagnosis is one of the primary reasons for the high mortality in DNM which ranging from 16% to 40% according to the most series [5,6].

Intravenous broad-spectrum antibiotic therapy alone is not efficient without adequate surgical drainage of the cervical and mediastinal collections, extensive debridement and excision of necrotic tissue, and wide mediastinopleural irrigation [6-14].

The most interesting aspect of the present case, not been reported before, is that our patient suffered of sub-acute mediastinal infection due to odontogen abscess treated with antibiotic therapy for three months. Then, an exacerbation of mediastinal infection is observed with concomitant presence of infected foot ulcer. In theory, the infection of foot ulcer may lead to septicemia which in addition with other risk factors as diabetes, and obesity may then cause the exacerbation of mediastinal infection.

In the present case, diagnosis of cervical infection with increasing infectious symptoms, and respiratory insufficiency is clinically obvious; yet, radiological studies show the presence of cervical abscess with diffuse mediastinal collections. Thus, the diagnosis of DNM is confirmed in agreement of diagnostic criteria of Estrera et al. [15]. These criteria include: (1] clinical manifestation of severe oropharyngeal infection; [2] demonstration of characteristic roentgenographic features of mediastinitis; [3] documentation of necrotizing mediastinal infection at operation or post-mortem examination or both; and [4] establishment of relationship between oropharyngeal infection and development of necrotizing mediastinal process.

In reported cases of DNM, successful surgical management involves a combination of cervical and mediastinal drainage with or without open thoracotomy. Endo et al. [16] proposed a classification scheme to facilitate management of DNM based on CT assessment of the extent of infection as follows: 1) Type I (or localized DNM) is an infection localised to the upper mediastinum above the tracheal bifurcation; 2) Type II A is defined an infection which involves the lower anterior mediastinum; 3) Type II B if the anterior and posterior lower mediastinum is involved. On the basis of CT findings, our case should be considered as Type II B; thus we support the use of an aggressive surgical treatment as combined cervicotomy and thoracotomy approach in order to evacuate all infected and necrotic tissue.

In the meta-analysis by Corsten et al [5], patients that receive combined cervicotomy and thoracotomy have a mortality of 19% versus 47% in patients receiving cervicotomy alone, which is a statistically significant difference. Marty-Ane et al. [6] achieve a relatively low mortality rate of 16.5% in their series of 12 patients by aggressively utilising thoracotomy. In their series, all patients that undergo both cervicotomy and thoracotomy survive. More recently, less invasive approaches successfully employing thoracoscopic or mediastinoscopic drainage have been reported.

Roberts et al [17] report the thoracoscopic management of a case of DNM with encapsulated mediastinal abscess; Gobien et al [18] propose CT-guided percutaneous drainage as a valuable alternative to surgical intervention in selected patients with mediastinal abscesses. However, in our case these procedures are inadequate for the spread of disease; thus, we attended an aggressive surgical treatment with left in place multiple drainage within mediastinum. They permitted the complete drainage of infection with resolution of disease.

## Competing interests

The authors declare that they have no competing interests.

## Authors' contributions

PL : conception and design, interpetration of data, given final approval of the version to be published. F. N.: acquisition of data, drafting the manuscript, given final approval of the version to be published. CC : acquisition of data, drafting the manuscript, given final approval of the version to be published. MD : acquisition of data, drafting the manuscript, given final approval of the version to be published. BA : acquisition of data, drafting the manuscript, given final approval of the version to be published. AG : acquisition of data, drafting the manuscript, given final approval of the version to be published. MV: acquisition of data, drafting the manuscript, given final approval of the version to be published. V.D.C.: critical revision, interpretation of data, given final approval of the version to be published

## Authors' information

PL: Resident in Department of Medicine and Surgery - University of Salerno. FN: Resident in Department of Medicine and Surgery - University of Salerno. CC: Resident in Department of Clinical Medicine and Surgery - University of Naples. MD: Aggregate Professor of Surgery in Department of Clinical Medicine and Surgery - University of Naples Federico II. BA: Associate Professor of Surgery in Department of Clinical Medicine and Surgery - University of Naples Federico II. AG: Assistant Professor of Pediatric Surgery - University of Salerno. MV: Associate Professor of Endocrinology - University of Salerno. VDC: Assistant Professor of Thoracic Surgery - University of Salerno.
